# Iranian future healthcare professionals' knowledge and opinions about rare diseases: cross-sectional study

**DOI:** 10.1186/s13023-022-02458-8

**Published:** 2022-09-29

**Authors:** Reza Jahanshahi, Amirreza Nasirzadeh, Mahan Farzan, Jan Domaradzki, Leila Jouybari, Akram Sanagoo, Mahour Farzan, Komeil Aghazadeh-Habashi, Ahmadreza Fallah Faraghe, Sadegh Bagheri, Marziyeh Samiee, Arina Ansari, Kimia Eskandari, Negar Namakkoobi, Fatemeh Soltanimoghadam, Hadi Mashali, Erfan Yavari, Saba Bay, Nafiseh Memaripanah, Elahe Meftah, Saeed Amanzadeh, Fatemeh Talati, Sasan Bahramzadeh

**Affiliations:** 1 Clinical Research Development Unit (CRDU), Sayad Shirazi Hospital, Golestan University of Medical Sciences, Gorgan, Iran; 2 Student Research Committee, Mashhad University of Medical Sciences, Mashhad, Iran; 3 Student Research Committee, Shahrekord University of Medical Sciences, Shahrekord, Iran; 4 Department of Social Sciences and Humanities, Poznan University of Medical Sciences, Poznan, Poland; 5 School of Nursing and Midwifery, Golestan University of Medical Sciences, Gorgan, Iran; 6 School of Nursing and Midwifery, Golestan University of Medical Sciences, Gorgan, Iran; 7 Student Research Committee, Tabriz University of Medical Sciences, Tabriz, Iran; 8 Student Research Committee, School of Nursing and Midwifery, Shahid Sadoughi University of Medical Sciences, Yazd, Iran; 9 Department of Nursing, School of Nursing and Midwifery, Gonabad University of Medical Sciences, Gonabad, Iran; 10 Department of Medical Laboratory Sciences, The School of Allied Medicine Sciences, Tehran University of Medical Sciences, Tehran, Iran; 11 Student Research Committee, School of Medicine, North Khorasan University of Medical Sciences, Bojnurd, Iran; 12 Student Research Committee, Torbat Heydariyeh University of Medical Sciences, Torbat Heydariyeh, Iran; 13 Student Research Committee, Rafsanjan University of Medical Sciences, Rafsanjan, Iran; 14 Department of Biology, Science and Art University, Yazd, Iran; 15 Student Research Committee, Shiraz University of Medical Science, Shiraz, Iran; 16 Student Research Committee, School of Nursing, Tehran University of Medical Sciences, Tehran, Iran; 17 Student Research Committee, Golestan University of Medical Sciences, Gorgan, Iran; 18 Student Research Committee, Semnan University of Medical Sciences, Semnan, Iran; 19 Students’ Scientific Research Center, Tehran University of Medical Sciences, Tehran, Iran; 20 Students Research Committee, Golestan University of Medical Sciences, Gorgan, Iran; 21 Department of Nutrition, Islamic Azad University, Sarvestan Branch, Sarvestan, Iran; 22 Student Research Committee, Hamadan University of Medical Sciences, Hamadan, Iran

**Keywords:** Rare diseases, Education, Awareness, Healthcare professionals

## Abstract

**Background:**

Rare diseases are a new global health priority, requiring evidence-based estimates of the global prevalence of diseases to inform public policymakers and provide a serious challenge to the healthcare system that must not be ignored. The purpose of this study is to investigate Iranian future healthcare professionals' knowledge and opinions about rare diseases.

**Results:**

A total of 6838 students responded to the questionnaire. Nursing and medical students had the highest participation. Almost 85% of participants rated their knowledge about rare diseases as poor or insufficient. While nearly 70 percent of participants took courses about rare diseases at university. Finally, 72.7% of future healthcare professionals did not feel ready to take care of a patient with a rare disease.

**Conclusion:**

The present study has indicated a gap in Iranian medical students’ knowledge of rare diseases. The researchers believe that health science policymakers should make a joint effort to improve knowledge about rare diseases. Including courses with regard to rare diseases would be of benefit to future healthcare professionals.

## Introduction

Rare diseases (RDs) are a new global health priority, requiring evidence-based estimates of the global prevalence of diseases to inform public policymakers. When a disease affects less than one in 2000 persons, it is considered as rare [1]. According to available information, 309 RDs have been diagnosed in Iran so far. Currently, around 3 million people with RDs have been identified in Iran and are receiving a variety of support services. Patients cared for by the Rare Diseases Foundation can benefit from a 50% discount on medicines by going to the Red Crescent. The Red Crescent is a non-governmental organization dedicated to humanitarian activities. In addition, it is a government-funded charity that also works to reduce the cost of treating patients with RDs. One of the most important missions of the Rare Diseases Foundation is to improve the lives of people with these diseases by raising awareness in the family and the community [2].

Rare diseases provide a serious challenge to the healthcare system that must not be ignored. Patients with RDs typically experience a lack of treatment alternatives, psychological stress, the financial burden in addition to the burden of disease [3]. Furthermore, these patients face challenges in obtaining a diagnosis of their illness, as it frequently takes 5–6 years to obtain an accurate diagnosis, and more than three different physicians are involved in the diagnosis process [4, 5]. Besides, due to a lack of knowledge about rare diseases and the challenges in properly diagnosing them, many patients with RDs would have visited multiple clinics or hospitals but only received symptomatic therapy [6].

One of the most common variables leading to the diagnostic odyssey is a lack of RD awareness and expertise among healthcare professionals [7]. Previous research has found that RD knowledge is typically considered inadequate by physicians and other health care professionals [8–10]. The majority of them believed their academic expertise was insufficient and unsuitable for diagnosing RDs in everyday practice [7, 11]. While physicians are responsible for managing the care processes of patients with RDs, the role of other health care professionals, including nurses and physiotherapists, is increasing. As a result, the need to increase knowledge and awareness of RD is evident among health care professionals [9]. Unfortunately, no study with a similar purpose was found in Iran and the knowledge of health care students is unknown. Due to the lack of basic information about the medical education curriculum in the field of rare diseases, we cannot predict the strength of the executive curriculum in the country's medical sciences universities.

Due to the importance of the subject, the purpose of this study is to investigate Iranian future healthcare professionals' knowledge and opinions about rare diseases.

## Methods

A cross-sectional survey-based study was conducted in Iran from September 2021 to January 2022 using convenience sampling. Our target population consisted of students of health care professionals who had completed two years of education and had access to the Internet. Data collection was carried out via online feedback from participants during the survey. An online questionnaire (Google Form) was administered which also contains the motivation letter explaining the purpose of the participant’s response, and the hyperlink to the questionnaire was eventually published by the authors in the virtual student groups of the medical universities of the whole country that had access. To control the answers, each researcher was given a three-digit number and a section was added to the online questionnaire to allow participants who received the questionnaire link from a trusted researcher could enter the researcher's three-digit code. This was done to ensure that the answers were genuine. Incorrect code answers were excluded from the study. The online questionnaire was designed in order that individuals can withdraw at any time. Over 10,000 students from all medical sciences universities participated in this study, and after removing those who did not meet the inclusion criteria or fill out the questionnaire incompletely, we reached 6838 eligible participants.

## Measures

### Demographic checklist

Includes: age, gender, marital status, the field of study, grade, duration of education, and domicile.

### Knowledge and attitudes of rare diseases questionnaire

The questionnaire used in the present study was developed by Domaradzki et al. based on the previous literature review and the aim of the study. The detailed description of the questionnaire and the method of its construction are explained elsewhere [9, 10].

In summary, the questionnaire consisted of 26 questions: the first questions concerned students' basic knowledge of RDs. Students were also asked to name RDs from a list that included twenty-eight diseases: 10 most common conditions and 18 RDs. The second section covered questions on organizational issues. The third section consisted of questions on students' awareness of RDs and their self-assessment knowledge and skills in the field of RDs.

The methodological procedures for the translation and cultural adaptation of the current questionnaire were employed as suggested by Beaton et al. according to a formal forward/backward translation protocol [12]. In the next step, some questions changed based on the cultural policies of Iran (questions: 6, 7, 12–18). The prepared version of the questionnaire was sent to 15 experts (Experts including nurse, physician, geneticist, sociologist, and physiotherapist) who were invited to give their opinion in order to improve the final version.

Content validity includes Content Validity Ratio (CVR) and Content Validity Index (CVI). Fifteen experts reviewed the questionnaire items and chose one of the essential, useful but not necessary, or unnecessary options [13]. According to the Lawshe table, the acceptance rate is 0.49 [14]. None of the phrases have been deleted in this section. After applying expert opinions and enriching the phrases, 15 other experts reviewed the questionnaire to evaluate CVI [15].

### Data analysis

Data were entered into SPSS software version 18 and reported in frequency, percentage, mean, and standard deviation. Differences in proportion were tested with the Chi-square test if any cells had an expected count smaller than 5. A statistical analysis of possible answers’ differences between all study groups was performed using the Kruskal–Wallis test. The level of significance was set at *p* < 0.05.

### Ethics

The study procedures were approved by the Clinical Research Development Unit (CRDU) of Sayad Shirazi Hospital of Golestan University of Medical Sciences and Ethical Review Board (IR.GOUMS.REC.1400.194). All participants entered the study with knowledge of the purpose of the study and informed consent and were informed that they could withdraw from the study at any time.

## Results

A total of 6838 people responded to the questionnaire. The majority of the participants were women (60.1%). Nursing and medical students also had the highest participation, 34.2% and 40% respectively. The demographics of the participants can be seen in Table 1. In the process of evaluating the content validity, no items were removed from the research, but changes were made in the form and richness of the words for better understanding. The CVR value was 0.5–0.9 for the items and 0.73 for the whole scale.

**Table 1:** Socio-demographic characteristics of students

Characteristics	N (%)
Gender	
Male	2731 (39.9)
Female	4107 (60.1)
Field of study	
Medical students	2732 (40)
Nursing students	2338 (34.2)
Midwifery students	637 (9.3)
Dentistry students	425 (6.2)
Physiotherapy students	111 (1.6)
Pharmacy students	490 (7.2)
Occupational and speech therapist students	105 (1.5)
Grade	
Bachelor degree	2807 (53.7)
Master degree	357 (5.2)
MD and DDS	3674 (53.7)
Marital status	
Single	6003 (87.8)
Married	835 (12.2)
Age (year)	22.7 ± 3.6
Duration of education (year)	5.6 ± 2.3
Domicile	
Under 10,000 inhabitants	423 (6.2)
10–50,000 inhabitants	1219 (17.8)
51–100,000 inhabitants	921 (13.5)
101–500,000 inhabitants	1271 (18.6)
Over 500,000 inhabitants	3..4 (43.9)
Have you ever met a person suffering from RD?	
Yes	3105 (45.4)
No	2154 (31.5)
I do not know	1579 (23.1)

Values are presented as n (%), mean ± standard deviation

Although almost all of the participants had heard of the term “rare disease” (96.4%), they were not sufficiently aware of its prevalence in the community (3.9%). only 8.5% correctly estimated the number of RD (Table 2). Students' knowledge about the number of RD patients in the world (6.2%), Asia (3.8%), and Iran (3.8%) was low. A quarter of future healthcare professionals knew the percentage of RDs that were genetic (24.8%). Most participants used the “I do not know” option when responding to the questions.

**Table 2:** Students' knowledge about rare diseases

Items	Medicals	Nursings	Others	*P*-value
	N (%)			
1. Have you ever heard the term 'rare diseases'?				
Yes	2636 (96.5)	2252 (96.3)	1701 (96.2)	0.884
No	96 (3.5)	86 (3.7)	67 (3.8)	
2. Rare disease is the one that affects less than:				
1 person in 1000	320 (11.7)	276 (11.8)	224 (12.7)	0.979
**1 person in 2000**	**107 (3.9)**	**89 (3.8)**	**68 (3.8)**	
1 person in 3000	91 (3.3)	73 (3.1)	55 (3.1)	
1 person in 5000	135 (4.9)	116 (5)	76 (4.3)	
1 person in 10,000	853 (31.2)	770 (32.9)	577 (32.6)	
I do not know	1226 (44.9)	1014 (43.4)	768 (43.4)	
3. What is the estimated number of rare diseases?				
100–500	196 (7.2)	187 (8)	129 (7.3)	0.082
1000–2000	282 (10.3)	237 (10.1)	160 (9)	
3000–5000	209 (7.7)	128 (5.5)	100 (5.7)	
**6000–8000**	**211 (7.7)**	**203 (8.7)**	**170 (9.6)**	
9000–1000	65 (2.4)	78 (3.3)	53 (3)	
Over 10,000	168 (6.1)	140 (6)	80 (4.5)	
I do not know	1601 (58.6)	1365 (58.4)	1076 (60.9)	
4. At what age group do rare diseases most frequently appear?				
Newborns	680 (24.9)	640 (27.4)	467 (26.4)	0.095
**Children**	**663 (24.3)**	**581 (24.9)**	**390 (22.1)**	
Adolescents	83 (3)	59 (2.5)	58 (3.3)	
Adults	135 (4.9)	101 (4.3)	76 (4.3)	
They are present in all age groups equally	404 (14.8)	324 (13.9)	273 (15.4)	
I do not know	767 (28.1)	633 (27.1)	504 (28.5)	
5. How many people suffer from rare diseases worldwide?				
10–15,000,000	142 (5.2)	145 (6.2)	103 (5.8)	0.592
50–75,000,000	213 (7.8)	143 (6.1)	121 (6.8)	
100–150,000,000	193 (7.1)	181 (7.7)	122 (6.9)	
200–250,000,000	126 (4.6)	128 (5.5)	90 (5.1)	
**300–350,000,000**	**172 (6.3)**	**149 (6.4)**	**100 (5.7)**	
Over 500,000,000	74 (2.7)	60 (2.6)	44 (2.5)	
I do not know	1812 (66.3)	1532 (65.5)	1188 (67.2)	
6. How many people in the Asia suffer from rare diseases?				
10–50,000,000	259 (9.5)	260 (11.1)	167 (9.4)	0.336
50–100,000,000	197 (7.2)	171 (7.3)	113 (6.4)	
100–200,000,000	138 (5.1)	106 (4.5)	84 (4.8)	
**200**–**250,000,000**	**74 (2.7)**	**49 (2.1)**	**40 (2.3)**	
250–300,000,000	112 (4.1)	95 (4.1)	57 (3.3)	
I do not know	1952 (71.4)	1657 (70.9)	1307 (73.9)	
7. How many people suffer from rare diseases in Iran?				
10–50,000	258 (9.4)	250 (10.7)	167 (9.4)	0.507
100–300,000	253 (9.3)	219 (9.4)	153 (8.7)	
300–500,000	201 (7.4)	154 (6.6)	114 (6.4)	
1–1,500,000	213 (7.8)	194 (8.3)	128 (7.2)	
**Over 2,000,000**	**96 (3.5)**	**95 (4.1)**	**72 (4.1)**	
I do not know	1711 (62.6)	1426 (61)	1134 (64.1)	
8. What is the most common cause of rare diseases?				
Infectious and bacterial	91 (3.3)	49 (2.1)	49 (2.8)	0.421
**Genetic**	**1911 (69.9)**	**1674 (71.6)**	**1244 (70.4)**	
Autoimmune	233 (8.5)	245 (10.5)	176 (10)	
Mitochondrial	39 (1.4)	27 (1.2)	23 (1.3)	
Environmental	50 (1.8)	42 (1.8)	30 (1.7)	
I do not know	408 (14.9)	301 (12.9)	246 (13.9)	
9. What percentage of rare diseases are of genetic origin?				
5–10%	270 (9.9)	227 (9.7)	180 (10.2)	0.911
20%	285 (10.4)	235 (10.1)	177 (10)	
50%	363 (13.3)	323 (13.8)	220 (12.4)	
**80%**	**676 (24.7)**	**585 (25)**	**432 (24.4)**	
100%	0 (0)	0 (0)	0 (0)	
I do not know	1138 (41.7)	968 (41.4)	759 (42.9)	

The correct answers to the questions are presented in bold

Participants selected rare diseases from a list of 28 diseases (Table 3). The most recognized RD were: Sickle cell anemia (30.2%), Marfan syndrome (28.6%), and Gaucher disease (28%). Least often were indicated Pompe disease (10.5%) and Osteogenesis imperfecta (9.9%). In contrast, the diseases most frequently confused with RD were Crohn’s disease (31.6%), Cerebral palsy (25.7%), and Glaucoma (21%).

**Table 3:** Which of the following diseases are considered to be rare in Iran?

Items	N (%)	*P*-value
**Fragile X syndrome**	**1365 (20)**	
Alzheimer’s disease	1499 (21.9)	
**Progeria**	**1006 (14.7)**	
**Duchenne muscular dystrophy**	**1466 (21.4)**	
Crohn’s disease	2164 (31.6)	
**Niemann-Pick disease**	**1029 (15)**	
**Huntington disease**	**1342 (19.6)**	
Cerebral palsy	1758 (25.7)	
**Marfan syndrome**	**1957 (28.6)**	
**Phenylketonuria**	**1165 (17)**	
Munchausen syndrome	438 (6.4)	
**Sickle cell anemia**	**2064 (30.2)**	
Down syndrome	1070 (15.6)	0.012
**Acromegaly**	**1077 (15.8)**	
**Osteogenesis imperfect**	**680 (9.9)**	
**Pompe disease**	**715 (10.5)**	
Schizophrenia	322 (4.7)	
**Mucopolysaccharidosis**	**1782 (26.1)**	
**Gaucher disease**	**1916 (28)**	
Halitosis	857 (12.5)	
**Haemophilia**	**1618 (23.7)**	
**Craniodiaphyseal dysplasia**	**1312 (19.2)**	
Glaucoma	1438 (21)	0.01
**Neurofibromatosis**	**1037 (15.2)**	
**Achondroplasia**	**874 (12.8)**	
**Cystic fibrosis**	**1482 (21.7)**	
Acquired immunodeficiency syndrome	1317 (19.3)	0.018
Fibromyalgia	532 (7.8)	

The correct answers to the questions are presented in bold

Future healthcare professionals' practical information about RD is shown in Table 3. Only 9.8% of participants were aware of the Iranian website (Rare Diseases Foundation of Iran) that provided information about RDs (https://radoir.org/). This is while about 59% of the participants were aware of the central register of RD patients in Iran and about 43% of them were aware of the existence of a national plan for RDs in Iran. Nursing students, compared to other students, mistakenly selected three diseases (Down syndrome, glaucoma, and Acquired immunodeficiency syndrome) as rare diseases (*P* < 0.05).

Students' perceptions of their knowledge of rare diseases are shown in Table 4. Almost 85% of participants rated their knowledge about rare diseases as poor or insufficient. While nearly 70 percent of participants took courses about rare diseases at university, a small number of them declared their knowledge because of the university courses. 60.3% of participants indicated that rare diseases are serious public health issues and, in their opinion, family physicians (49.3%) and geneticists (64.6%) should be trained specifically in rare diseases. Finally, 72.7% of future healthcare professionals did not feel ready to take care of a patient with a rare disease.

**Table 4:** Students' knowledge about healthcare system for RD patients

Items	Medicals	Nursings	Others	*P* value
	N (%)			
11. What percentage of rare disease can be treated with drugs?				
0%	212 (7.8)	168 (7.1)	121 (6.8)	0.84
**5%**	**326 (11.9)**	**278 (11.9)**	**220 (12.4)**	
10%	235 (8.6)	234 (10)	161 (9.1)	
15%	168 (6.1)	151 (6.5)	109 (6.2)	
20%	183 (6.7)	171 (7.3)	123 (7)	
50%	85 (3.1)	54 (2.3)	57 (3.2)	
I do not know	1523 (55.7)	1283 (54.9)	977 (55.3)	
12. When is rare disease day celebrated?				
January 26	79 (2.9)	59 (2.5)	51 (2.9)	0.77
**February 26**	**489 (17.9)**	**414 (17.7)**	**328 (18.6)**	
March 26	67 (2.5)	68 (2.9)	50 (2.8)	
April 26	94 (3.4)	90 (3.8)	58 (3.3)	
I do not know	2003 (73.3)	1707 (73)	1281 (72.5)	
13. The Iranian non-governmental patient’s organization in the field of rare diseases is:				
Ghoghnoos	132 (4.8)	113 (4.8)	79 (4.5)	0.25
**Iranian Rare Diseases Foundation**	**575 (21)**	**529 (22.6)**	**366 (20.7)**	
Zanjireh Omid International Charity	185 (6.8)	187 (8)	109 (6.2)	
Rare Disease Protection O	292 (10.7)	242 (10.4)	202 (11.4)	
I do not know	1548 (56.7)	1267 (54.2)	1012 (57.2)	
14. What is the name of the Iranian website providing information about RD and orphan drugs?				
**Radoir**	**255 (9.3)**	**229 (9.8)**	**185 (10.5)**	0.46
NORD	173 (6.3)	137 (5.9)	103 (5.8)	
EURORDIS	59 (2.2)	38 (1.6)	39 (2.2)	
RARE	167 (6.1)	167 (7.1)	99 (5.6)	
Orphanet	65 (2.4)	45 (1.9)	33 (1.3)	
Global Genes	28 (1)	32 (1.4)	23 (1.3)	
I do not know	1985 (72.7)	1690 (72.3)	1286 (72.7)	
15. Is Iran a member of the world organization for rare diseases?				
**Yes**	**1088 (39.8)**	**923 (39.5)**	**672 (38)**	0.45
No	186 (6.8)	187 (8)	137 (7.7)	
I do not Know	1458 (53.4)	1228 (52.5)	959 (54.2)	
16. Is there a central register of RD patients in Iran?				
**Yes**	**1499 (54.9)**	**1253 (53.6)**	**999 (56.5)**	0.17
No	154 (5.6)	161 (6.6)	88 (5)	
I do not know	1079 (39.5)	924 (39.5)	681 (38.5)	
17. Are orphan drugs reimbursed in Iran?				
Yes	800 (29.3)	608 (26)	517 (29.2)	0.007
**Yes, some**	**1139 (41.7)**	**977 (41.8)**	**718 (40.6)**	
No	0 (0)	0 (0)	0 (0)	
I do not know	793 (29)	753 (32.2)	533 (30.1)	
18. Is there a national plan for rare diseases in Iran?				
**Yes**	**795 (29.1)**	**681 (29.1)**	**561 (31.7)**	0.117
No	490 (17.9)	463 (19.8)	284 (16.1)	
I do not know	1447 (53)	1194 (51.1)	923 (52.2)	

The correct answers to the questions are presented in bold

None of the demographic items had a significant relationship with students' knowledge and attitudes (*P* > 0.05). Students' self-assessment of their knowledge about RD is shown in Table 5.

**Table 5:** Students' self-assessment of their knowledge about RD

Items	Medical s	Nursing s	Other s	*P* value
	N (%)			
Do RD constitute a serious public health issues?				
Absolutely yes	820 (30.01)	668 (28.57)	516 (29.18)	0.15
Yes	850 (31.11)	700 (29.9)	570 (32.23)	
No	281 (10.2)	239 (10.2)	198 (11.19)	
Definitely no	66 (2.41)	51 (2.18)	47 (2.65)	
I do not now	715 (26.17)	680 (29.08)	437 (24.71)	
Which physicians should be uniquely trained in RD?				
Family physician	1356 (49.6)	1133 (48.5)	881 (49.8)	–
Pediatrician	932 (34.1)	842 (36)	602 (34)	
Neurologist	497 (18.2)	411 (17.6)	329 (18.6)	
Geneticist	1755 (64.2)	1547 (66.2)	1116 (63.1)	
Psychiatrist	453 (16.6)	402 (17.2)	310 (17.5)	
Immunologist	739 (27)	629 (26.9)	508 (28.7)	
Have you had any classes about rare disease during your studies?				
Yes	1879 (68.8)	1629 (69.7)	1238 (70)	0.63
No	594 (21.7)	491 (21)	383 (21.7)	
I do not know	259 (9.5)	218 (9.3)	147 (8.3)	
How would you rate your knowledge about rare diseases?				
Very good	51 (1.9)	45 (1.9)	29 (1.6)	0.91
Fair enough	366 (13.4)	279 (11.9)	223 (12.6)	
Insufficient	1197 (43.8)	1072 (45.9)	791 (44.7)	
Very poor	1118 (40.9)	942 (40.3)	725 (71)	
Would you like to broaden your knowledge about rare diseases?				
Yes	2213 (81)	1865 (79.8)	1403 (79.4)	0.44
No	300 (11)	292 (12.5)	231 (13.1)	
I do not know	219 (8)	181 (7.7)	134 (7.6)	
Do you think that there should be a mandatory course on rare diseases in medical curricula?				
Definitely yes	584 (21.4)	528 (22.6)	382 (21.6)	0.60
Rather yes	1378 (50.4)	1168 (50)	898 (50.8)	
Rather not	423 (15.5)	353 (15.1)	258 (14.6)	
Definitely not	188 (6.9)	166 (7.1)	135 (7.6)	
I do not know	159 (5.8)	123 (5.3)	95 (5.4)	
Where do you get your knowledge about RD from?				
Mandatory courses at the university	663 (24.3)	590 (25.2)	462 (26.1)	–
Faculty courses at the university	193 (7.1)	135 (5.8)	142 (8)	
Scientific literature and research	654 (23.9)	533 (22.8)	448 (25.3)	
Scientific conferences, symposia	313 (11.5)	231 (9.9)	180 (10.2)	
Internet	1517 (55.5)	1332 (57)	954 (54)	
I do not search for such information	546 (20)	442 (18.9)	344 (19.5)	
Do you feel prepared for caring over a patient with a rare disease?				
Definitely yes	164 (6)	123 (5.3)	91 (5.1)	0.24
Rather yes	605 (22.1)	508 (21.7)	382 (21.6)	
Rather not	858 (31.4)	710 (30.4)	544 (30.8)	
Definitely not	814 (29.8)	751 (32.1)	552 (31.2)	
I do not know	291 (10.7)	246 (10.5)	199 (11.3)	

## Discussion

According to the literature review, no studies were found in Iran examining the knowledge of future healthcare professionals about RD. Due to the growing understanding of the medical community about genetically based diseases, there is a need for educational programs in the area of RD in particular. As a result, future healthcare professionals often do not receive the necessary training in RD [16].

Consistent with previous studies [16–19], the results of the present study showed that future healthcare professionals receive little education about RD. Although almost all of the participating students were familiar with RD and most believed that RD was due to genetic factors, they had little knowledge of the epidemiology of RD. Many students have used the "I do not know" option and the problem of insufficient education is evident during their studies. In Iranian medical universities, basic sciences including biochemistry, physiology as well as genetics are taught in the first year. Regarding the major, it may continue or not. Numerous RDs such as PKU, CF, Huntington’s disease, and sickle cell disease are included in genetics courses. In addition to the diseases, diagnostic tests and major clinical manifestations are also taught. We hypothesize that medical sciences students (MSSs) learn the knowledge of RDs randomly and casually. The premise is according to the following reasons: 1) while most students were aware of their knowledge deficits, they had an unsatisfactory level of information on RDs. 2) RDs are not taught as a separate subject and in a systematic comprehensive form. Rather, it is considered a subset of genetics. So all of MSSs are deprived of training in relation to RDs. In line with our findings, previous studies [9, 20] authenticate the fact that the retention of MSSs’ knowledge and skills is compromised. Many studies, like our results, addressed the unfavorable level of knowledge of the students [10, 16, 17]. Using similar tools, Domaradzki et al. [9] pointed out that the students' knowledge was insufficient. The vast majority of respondents were also aware of their lack of knowledge. In their study, medical students were significantly better informed about RD than other students. However, the findings of our study showed that there was no significant difference in the knowledge and attitude of nursing, medical, and the other students. The results of a study conducted in Kazakhstan show that medical students and physicians suffer from inadequate education in rare diseases. They suggested an immediate revision of medical education standards [21].

In the present study, some of MSSs have selected the following diseases as RDs mistakenly: Crohn’s disease (31.6%), Cerebral palsy (25.7%), and Glaucoma (21%). The above-mentioned diseases are diseases which are well discussed in the classes. Lack of recognition of rare diseases from other diseases indicates that misdiagnosis can delay treatment. More interestingly, in high school education, some basic information about genetic disorders and RDs is already present, therefore this problem is rooted in an educational system which is consistent with Williams’ findings [22]. These results indicate that the medical sciences curriculum is likely to be disrupted and inappropriate.

Moreover, there is no specific course, guideline, or recommendations at Iranian universities pertaining to RDs. Our medical education system usually focuses on more prevalent disorders and prepares students for facing conventional diseases rather than rare ones. More studies in this field are needed to determine whether an elective or mandatory course on RDs should be included in the medical education curriculum.

Additionally, preparing a comprehensive content including multidimensional information about RDs like prevalence, incidence, the relevance of RDs to everyday medical care, early detection, potential treatment strategies, and the last but not least, the challenges that healthcare workers face during admission of RD patients is necessary. Healthcare workers have to know sources of information and support for RD patients to help RD patients’ families and caregivers.

McKay [23] suggests that teaching programs should not focus on any particular RD. There are an estimated 6000–8000 different types of RDs and focusing on all RDs is impossible. Trying to raise public awareness about cursory knowledge of the prevalence and incidence of RDs would be of profound importance. Pisklakov acknowledges that health professionals’ false beliefs may interfere with their attitude and disrupt the situation. This is of key importance because health professionals’ false beliefs in their knowledge and skills make it difficult to change the situation of patients with rare diseases [24] Alawi et al. [25] suggest using RDs as a teaching model to transfer the basic sciences and clinical practice to students.

The findings of our study showed that there was no significant difference in the knowledge and attitude of nursing, medical, and the other students.

The present study has limitations, the most important of which is the content validity of the questionnaire. The items used in the questionnaire used were made in accordance with the conditions of Poland and European policies. Therefore, it cannot adequately cover all the required aspects of Iran's health policy. It is proposed to conduct more detailed studies to create a questionnaire according to Iran's health policy. The questionnaire may not effectively measure numerous concepts such as diagnosis, care, and treatment. It is suggested that future studies explore other concepts in the curricula of universities of medical science.

In conclusion, the present study shows that, according to the participants' answers, the existence of curricula on rare diseases is not enough for them and they do not feel ready to care for patients with rare diseases. We have shown that a fundamental problem exists and we suggest that future researchers look into the causes of this problem so that steps can be taken to improve the current situation.

## Conclusion

The present study has indicated a gap in Iranian medical students’ knowledge of RDs. The researchers believe that health science policymakers should make a joint effort to improve knowledge about RDs. Including courses with regard to RDs would be of benefit to future healthcare professionals, the importance of pharmaceutical education on orphan drugs which could be included in the medical curricula; the need to establish closer collaboration with other neighboring countries, the role of machine learning and artificial intelligence that can support the decision process and overcome barriers that affect the diagnostic odyssey and the role of telemedicine and telepharmacy services in providing RD patients the opportunity to continue treatment.

## Data Availability

All data generated or analyzed during this study are included in this published article.
